# Ultrasound-Assisted Slightly Acidic Electrolyzed Water in Aquatic Product Sterilization: A Review

**DOI:** 10.3390/foods11233863

**Published:** 2022-11-30

**Authors:** Chenchen Zhang, Jing Xie

**Affiliations:** 1 College of Food Science and Technology, Shanghai Ocean University, Shanghai 201306, China; 2 National Experimental Teaching Demonstration Center for Food Science and Engineering, Shanghai Ocean University, Shanghai 201306, China; 3 Shanghai Engineering Research Center of Aquatic Product Processing and Preservation, Shanghai 201306, China; 4 Shanghai Professional Technology Service Platform on Cold Chain Equipment Performance and Energy Saving Evaluation, Shanghai 201306, China; 5 Collaborative Innovation Center of Seafood Deep Processing, Ministry of Education, Dalian 116034, China

**Keywords:** ultrasound, slightly acidic electrolyzed water (SAEW), microorganisms, aquatic products, sterilization

## Abstract

Ultrasound has been confirmed as the propagation process of mechanical vibrations in a medium, with a frequency significantly higher than 20 kHz. Moreover, it has an effect of sterilization on foods. In general, ultrasonic sterilization medium is manifested as a liquid. Ultrasonic treatment technology has certain advantages in aquatic product processing. It is noteworthy that this technology will have better effects of sterilization if used in combination with other treatment methods. Slightly acidic electrolyzed water (SAEW) is characterized by high-efficiency broad-spectrum sterilization operation, low cost, and environmental protection, among other properties, and has a positive effect on aquatic product sterilization and preservation. Selecting acidic electrolyzed water with a low concentration coupled with low-power ultrasonic waves for combined sterilization exerts a more potent sterilization effect, and acidic electrolyzed water combined with ultrasonic sterilization is expected to be a potentially environment-friendly alternative. In this study, the sterilization mechanisms of ultrasonic and SAEW methods used both individually and as a synergistic treatment, the effect on microbial growth, and the research progress of the application of the combined effect in the sterilization and refrigeration of aquatic products are reviewed. Furthermore, this study looks forward to the future development trend, with a view to its application in aquatic products, while providing a reference for research and application in the field of processing and safety.

## 1. Introduction

Nutrients (e.g., proteins, vitamins, and minerals) abound in aquatic products [1]. The action of microorganisms and endogenous or exogenous enzymes account for the gradual deterioration of aquatic products during storage [2,3]. Food safety issues arising from microorganisms have always been a social hotspot that arouses public concern [4,5]. Currently, commercial biocides (e.g., chlorine gas, peroxide combinations, and quaternary ammonium compounds) are employed throughout the food supply chain [6]. Commonly used oxidation fungicides are chlorine gas, hydrogen peroxide, and so on, which mainly act through the metabolism of enzymes and bacteria in the body’s oxidation process to achieve the purpose of sterilization. The above biocides have certain limitations, including a potential danger to people or the environment, pesticide residues, and inferior food quality. Accordingly, research and development of safe and effective sterilization methods for aquatic products have aroused extensive attention [7].

Ultrasound refers to a mechanical wave with a significantly small wavelength, which is almost always less than 0.02 m (meters) in the air. It cannot exist in a vacuum (e.g., space), and its existence and reproduction are dependent on a medium. Ultrasound travels farther in water than in air, whereas it is very easy to lose and disperse in the air due to its small wavelength [8]. Although ultrasound does not travel as far as audible sound and infrared waves, short wavelengths make ultrasound easier to obtain anisotropic sound energy, which have been widely applied for cleaning, gravel, sterilization, and other purposes [9]. Longitudinal waves are formed during ultrasonic treatment when high-intensity ultrasonic waves propagate through liquid media, thus resulting in zones of alternate compression and expansion [10]. The above changes in pressure are prone to cavitation, thus resulting in the creation of tiny bubble nuclei in the medium. The micro-bubble core exhibits high temperatures and pressures during adiabatic shrinkage and collapse, which kills certain microorganisms in the liquid and inactivates viruses.

SAEW was electrolyzed with dilute hydrochloric acid of 2~6%, and its pH value was 5.0~6.5. It contained a concentration of accessible chlorine (HClO) of 10,000 to 30,000 mg/L, was colorless and odorless, and had a high bactericidal action. In the food field, compared with the currently commonly used bactericides such as sodium hypochlorite or alcohol, it can ensure safety, and has the characteristics of reducing cost and reducing environmental load [11,12]. Compared with sodium hypochlorite or alcohol, SAEW exhibits the characteristics as follows. It will not result in chlorine (Cl_2_) residues in food, it is not required to be rinsed with clean water to save water, and there is little need to worry about mistakes in its usage as problems will not be caused. SAEW has been proved to be effective in destroying MRSA (Staphylococcus aureus), *Escherichia coli*, norovirus, and influenza virus.

In this study, the sterilization mechanism of ultrasonic waves and SAEW used both separately and synergistically was investigated regarding its effect on microbial growth and the application of the combined effect in the sterilization of aquatic products was reviewed. In addition, the development trend for the foreseeable future was forecasted. The research and application in aquatic product processing and safety provide a reference, and also novel ideas for the expansion of sterilization and refrigeration technology.

## 2. Ultrasonic Waves

Ultrasonic refers to sound waves with a frequency greater than 20 kHz. It has a short wavelength and a high frequency. In addition to its good directionality and penetrating force, it has the potential to cause cavitation and several specific effects (e.g., mechanical, thermal, and chemical effects) [13]. The cavitation produced by ultrasonic waves has been confirmed as the principal reason for their bactericidal impact [14,15].

### 2.1. Ultrasound-Assisted Mechanism

**Figure 1:**
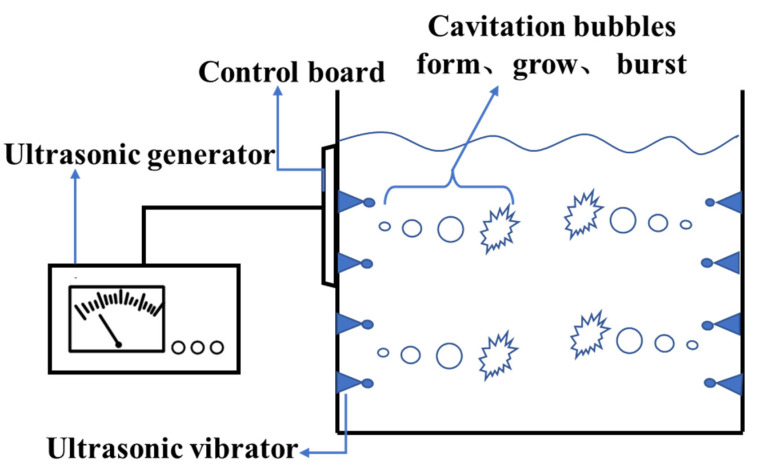
Reaction principle of ultrasound [16].

Figure 1 presents the working principle of ultrasonic treatment technology [16]. Pictured are an ultrasonic generator, ultrasonic vibrator, and other components. The ultrasonic generator, also known as the ultrasonic drive power, electronic box, or ultrasonic controller, is an important part of a high-power ultrasonic system. The function of the ultrasonic generator is to convert the mains electricity into high-frequency AC electrical signals matched with the ultrasonic transducer and drive the ultrasonic transducer’s operation. The ultrasonic vibrator generates radial vibration to the rod body through a transducer with high power and amplitude, and generates ultrasonic waves evenly across 360° near the vibrator. Therefore, the direction of use is flexible.

When ultrasound acts on food, four effects are exerted: cavitation effect, mechanical effect, thermal effect, and free radical effect. To be specific, the first three effects are physical effects, and the free radical effect is a chemical effect [17,18].

The cavitation effect is explained as follows: a considerable number of small bubbles are produced when the ultrasonic wave is acting on the liquid [19]. One reason for this is that a tensile stress appears in the liquid to form negative pressures. The gas that was previously dissolved in the liquid became saturated as the pressure drops, and the liquid is freed and becomes a small bubble. Another reason is that the liquid is “torn” by the strong tensile stress into a hole called a void. Another type of gas formed from liquid vapor, or soluble substances dissolved in liquid in the cavity, may even create vacuums. Small bubbles formed by the effect of hollowing are constantly growing or may suddenly burst. When the surrounding liquid suddenly rushes into the bubbles, higher pressure follows. The factors influencing hole formation include intense ultrasonic irradiation, shock during explosion, high speed fluid impulse friction, or intense chemical reaction. The internal friction accompanied by the effect of hollowing can form a charge, and the phenomenon of glowing is due to discharge in bubbles. The techniques for ultrasound treatment in liquid are largely correlated with the cavitation effect [20]. The mechanical effect of an ultrasonic wave can facilitate liquid emulsification and gel liquefaction, as well as solid decentralization. The action of mechanical power causes minute particles suspended in the ultrasonic fluid medium to condense at the section where waves are formed in the medium, thus resulting in a periodic accumulation in space.

The mechanical effect of ultrasound is the most basic primary effect of ultrasound, regardless of the intensity of such effects. The mechanical effect of an ultrasonic wave comes from the wave moving forward in the medium, which is called the mechanical effect in the traveling wave field. In addition, the mechanical effect caused by the reflection of ultrasonic waves in the medium is called the mechanical effect in the standing wave field [21]. The thermal effect is constantly accompanied by the mechanical effect [22]. The thermal shock of an ultrasonic wave refers to the transformation of some energy when the ultrasonic wave passes through the medium. Its energy is partially converted into heat energy through the process of friction and heat conduction, thus leading to the increased local temperature of the medium. The temperature tends to increase during the irradiation, and the temperature increase and the radiation time are proportional. When the temperature increases to a certain degree, the rate of increased temperature begins to decrease [23]. Ultrasonic waves are capable of facilitating the hydrolysis, decomposition, and polymerization of a considerable number of chemicals. Moreover, ultrasonic waves also have an obvious effect on photochemical and electrochemical processes. After ultrasonic treatment of aqueous solutions of a wide variety of amino acids and other organic materials, the characteristic absorption bands disappear and show uniform general absorption, thus suggesting that the molecular structure has been changed by cavitation. The free radical effect refers to the chemical reaction of free radicals through the breaking of chemical bonds [24]. A free radical reaction is one of a variety of chemical reactions in which free radicals participate. The outer shell of the electron shell of the free radical has an unpaired electron, which has a strong affinity for adding a second electron, so it can act as a strong oxidant. The liquid medium is changed to generate free radicals due to the collapse of the cavitation bubbles, thus leading to a free radical effect occurring.

**Figure 2:**
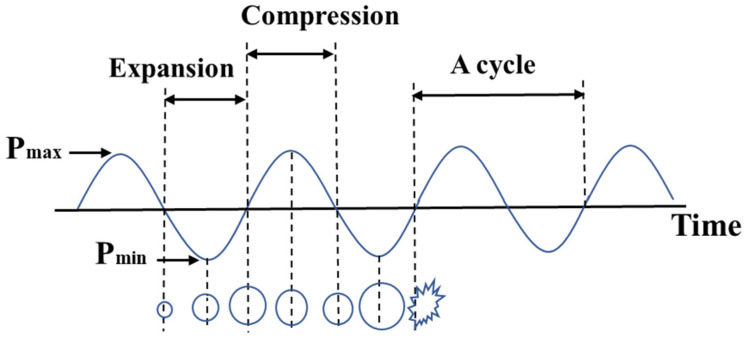
Cavitation effect process of ultrasonic wave [25].

The cavitation effect primarily accounts for the ultrasonic sterilization effect. Figure 2 depicts a schematic diagram of ultrasonic cavitation effects. When the ultrasonic wave penetrates the medium, the sound pressure in the whole medium undergoes periodic changes of positive and negative pressure [25]; the bubbles are cavitation bubbles; when the pressure is suddenly reduced, it causes the microbubbles to burst and release energy [26]. This local and instantaneous release of energy seriously damages or even ruptures the cell wall and cell membrane. Moreover, the cytoplasm shrinks, the cytoplasmic structure is disordered, and the organelle structure is destroyed. In addition, cell content proteins and nucleic acids are leaked, and open and irreversible microbial cell structures are produced [27]. The destruction or breakdown of cellular structures causes the death of microorganisms, thus extending the shelf life of foods [28,29,30]

### 2.2. The Impact of Ultrasound on Microorganisms

Factors affecting the inactivation of microorganisms by ultrasonic waves include ultrasonic parameters (such as frequency, power, amplitude, etc.), the medium in which the bacteria are located, the time of ultrasonic action, and the types of microorganisms. Most microorganisms are completely or selectively eliminated [31,32,33].

LIN et al. [34] have suggested that the inhibitory effect on *Escherichia coli* O_157_:H_7_ reaches 68.91% under the condition of ultrasonic power of 100 W and the power density of 50 W/cm^2^ for 420 s, and the ultrasonic treatment makes the *E. coli* O157:H7 cell membrane permeable. As a result, the antibacterial activity is significantly increased, the integrity of the cell membrane is destroyed, and proteins and DNA are leaked, and thus the metabolism-related enzyme activities are reduced. An in-depth study on the antibacterial mechanism has suggested that ultrasound is capable of producing free radicals, thus increasing intracellular oxidative stress, blocking the hexose phosphoric acid pathway, and inhibiting energy metabolism [35].

Wu et al. [36] investigated the impact of ultrasonic damage on cell walls and membranes at three different intensities (10, 24, and 39 W/cm^2^). Some researchers have suggested that at moderate acoustic intensities (10 W/cm^2^), the release of cell wall polysaccharides is faster than that of intracellular proteins, with a reversed result at higher sonic levels (24 and 39 W/cm^2^). The above results reveal that the ultrasonic damage target shifted when the ultrasonic treatment intensity shifted between low and high [37]. The collapse of the cell wall is the first step in ultrasonic disruption of yeast cells, followed by low-intensity damage to the cell membrane. The above findings can create new possibilities for cell disruption and microorganism inactivation [38]. The same study suggested that high temperatures disrupt cell walls and thermally adhere to intracellular proteins.

Pedrós-Garrido et al. [39] treated salmon with ultrasound at a frequency of 30 kHz and a power of 51.41 W/L for 2700 s. They suggested that the numbers of psychrophilic and mesophilic bacteria are reduced by log 1.5 and 1.1, respectively. Ultrasound has a bactericidal effect. It is capable of changing the adhesion of microbial biofilm to aquatic products through mechanical vibration, inhibiting its adhesion to the surface of aquatic products, as well as reducing bacteria [40]. Gao et al. [40] investigated the performance of bacterial suspension activity under experimental conditions (including high intensity and low frequency ultrasound). *Enterobacter aerogenes* and *Bacillus subtilis* are destroyed through ultrasonic treatment (up to log 4.5 reduction). However, strains of Staphylococcus spp. do not change in any way. Furthermore, *E. aerogenes* suspension is more responsive to ultrasonic waves at the exponential development stage than at later stages. The existing study has suggested that microbes with larger, softer capsules are more resistant to ultrasonic inactivation methods. The structure of bacterial cells fundamentally accounts for their resistance to ultrasonic deactivation.

Evelyn and Silva [41] increased thermal inactivation of *Clostridium perfringens* spores in beef slurry using power ultrasound. Spore thermal resistance in beef pulp remained unchanged at 353.15 K for 600 s. The same heat shock, paired with a 60 s ultrasound treatment, reduced *C. perfringens* spores in beef slurry by half. On that basis, they proposed a method of promoting *C. perfringens* spore heat inactivation, thus leading to shortened food processing times and improved food quality.

However, existing research has suggested that ultrasonic treatment under certain conditions is capable of reducing the growth of microorganisms in food, prolonging the food’s shelf life, without affecting, and in some cases improving, the quality of food [42]. However, when ultrasound is used alone, the bacteriostatic rate often does not meet the specified requirements. Although the bacteriostatic rate can be improved by increasing the ultrasonic intensity, energy waste will be caused.

## 3. Slightly Acidic Electrolyzed Water

As a novel type of bactericide, SAEW has the characteristics of low pH, high redox potential, and effective chlorine, which can kill bacteria quickly and widely [43]. In the field of food, compared with the commonly used sodium hypochlorite or alcohol fungicides, it has the characteristics of ensuring safety, reducing cost, and reducing environmental load [44]. The germicidal effect of slightly acidic electrolytic water is affected by the available chlorine mass concentration, pH value, and redox potential [45,46].

### 3.1. The Impact of Slightly Acidic Electrolyzed Water on Microorganisms

SAEW is a potential alternative to antibacterial detergents and is considered an environmentally beneficial disinfection method with the advantage of reducing the impact of residual chlorine on human health and safety compared with other disinfectants [47]. The antibacterial action of SAEW is mostly due to the ability of HClO to cause oxidative damage to biomolecules. Microorganisms in food can be significantly reduced by using SAEW in combination with other germicidal or mechanical forces in the washing process [48,49].

Liu et al. [50] have suggested that SAEW exhibits strong antibacterial activity against tested microorganisms, which is significantly correlated with the available chlorine concentration (ACC) of SAEW. The mortality rate of *S. putrefaciens* and *S. saprophyticus* reaches 96% and 85%, respectively, at the ACC value of mildly acid electrolytic water of 60.0 mg/L. The results of scanning electron microscopy (SEM) indicated that SAEW disrupts cell morphology and structure. Furthermore, the formation of ROS is influenced by SAEW. Ultimately, the accumulation of ROS causes a cascade of physiological abnormalities within the cell, leading to bacterial death. Hao et al. [51] studied the efficacy of SAEW and AEW against three test bacteria (e.g., *Escherichia coli*, *Staphylococcus aureus*, and *Bacillus subtilis*). The result of experiments has suggested that the disinfection effect on the three studied strains rapidly diminished with increasing dilution of AEW and SAEW, as indicated by the existing research. When AEW is diluted 17 times, the number of live E. coli, *Staphylococcus aureus*, and *Bacillus subtilis* fell to 7.84, 7.34, and 7.90 log10 CFU / ml, respectively. Survival rates for *E. coli*, *S. aureus*, and *Bacillus subtilis* decreased to 7.96, 6.45, and 7.89 log10 CFU /mL, respectively, at the dilution of 47 times. In addition, the results demonstrate that SAEW is more effective against the above strains than AEW in terms of antibacterial efficacy. However, even when SAEW is diluted more than AEW, its disinfection efficiency is the same. Xuan et al. [52] suggested that SAEW-ice is much more beneficial to enhance squid preservation than TW-ice during storage. After SAEW ice treatment, the bacterial count decreases by 1.46 ± 0.10 log10 CFU/g, and microbial growth remains relatively slow during storage. SAEW-ice extends the shelf life of seafood and improves its safety and quality by serving as a preservative and disinfectant. Zhang et al. [43] investigated the sporangial inactivation effect of slightly acidic electrolyzed water (SAEW) at different available chlorine concentrations (ACC, 20, 60, and 100 mg/L), as well as the spore structure changes, mutagenesis, and inner membrane (IM) properties. The findings show that SAEW treatment causes damage to the spore surface and IM, and loss of core content due to rupture. Surviving SAEW-treated spores do not produce mutants. SAEW significantly attenuates spore viability media in high salinity environments. Spores treated with SAEW were induced to germinate with L-alanine or inosine and stained with propidium iodide (PI), but lysozyme addition failed to restore them [53]. Furthermore, SAEW treatment inhibits spore germination during germination induction. The above findings suggest that SAEW inactivates spores mainly by destroying the spore IM.

Through the above studies, it is found that microorganisms, such as *Escherichia coli* and *Salmonella*, are significantly reduced by SAEW treatment. Spores of botrytis cinerea, *Botrytis cinerea*, *Bacillus anthracis*, and *Phytophthora capsicum* cannot be cultured following treatment. Therefore, SAEW has strong broad-spectrum antibacterial and fungal activities.

### 3.2. Slightly Acidic Electrolyzed Water Mechanism

The physicochemical properties of fungicides (e.g., water solubility, to a large extent, and stability regarding biological and abiotic degradation processes) affect the actual effect of fungicides [54,55]. Pure water and electrolyte are pumped into the electrolytic cell. Under the action of direct current, the negatively charged ions (chloride and hydroxide) move to the anode and lose electrons in the anode while forming oxygen (O_2_), chlorine gas (Cl_2_), hypochlorite ion (ClO_-_), hypochlorous acid (HClO), and hydrochloric acid (HCl). Furthermore, positively charged ions (hydrogen and sodium) move to the cathode where they gain electrons to form hydrogen gas (H2) and sodium hydroxide (NaOH).

The chemical reactions:

at anode: 2NaCl→Cl_2_(g) + 2e^−^ + 2Na^+^, 2H_2_O(l)→4H^+^(aq) + O_2_(g) + 4e^−^,

Cl_2_ + H_2_O(1)→HCl + HOCl

at cathode: 2H_2_O(l) + 2e^−^→2OH^−^(aq) + H_2_(g),

2NaCl + 2OH^−^→2NaOH + Cl^−^

**Figure 3:**
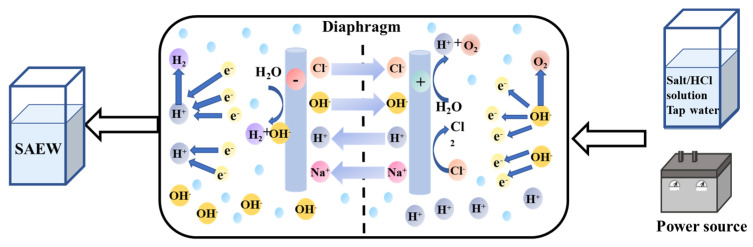
The schematic illustration for slightly acid electrolyzed water application [56].

Figure 3 presents a schematic for the application of SAEW [56]. Slightly acidic electrolytic water is made by low-voltage electrolysis. In the anode, 2HCl→ H^+^ + Cl^−^, which in turn reacts with OH^-^ to form hypochlorous acid (HOCI) and hydrochloric acid (HCl). Slightly acidic electrolytic water occurs after the REDOX reaction to generate water. The raw materials do not contain salt; thus, even after drying, the water will not cause harm related to the concentration and crystallization of salt.

In a membraneless chamber, electrolyzing 2–6% dilute hydrochloric acid produces somewhat acidic electrolyzed water with a pH of 5.0–6.5. There are three forms of chlorine in SAEW: chlorine gas (Cl_2_), hypochlorite ion (−OCl), and hypochlorous acid (HClO). The strongest effect, the bactericidal ability of HClO, is about 80 times that of −OCl [57]. It is an aqueous solution with hypochlorous acid as the main component with bactericidal effect [58]. Because of its minimal running expenses and excellent antibacterial action [46], it has been frequently used to sterilize goods (e.g., fruits [59], vegetables, and meat). When electrolytic water is used, microorganisms (e.g., *Escherichia coli*, *Staphylococcus aureus*, *Salmonella, vibrio parahaemolyticus* and *Bacillus cereus*) all exhibit the effects of considerable antibacterial activity [60]. SAEW exhibits bactericidal properties by attacking multiple targets of microbial cells (e.g., cell walls, extracellular membranes, and intracellular components) [61]. The surface shape of the microbial cells changes from smooth, continuous, and bright to rough, shrunken, and decomposed after the cells are treated with SAEW. The antibacterial activity of SAEW is primarily due to the potential of hypochlorous acid in causing oxidative damage to biomolecules [62]. Microorganisms in foods can be significantly reduced using slightly acidic electrolytic water during the washing process in combination with additional disinfectant or mechanical forces [63].

## 4. Synergistic Treatment of Ultrasonic Waves Combined with Slightly Acidic Electrolyzed Water

Sonication alone (at room temperature and ambient pressure) has been shown to be inefficient for inactivating several bacterial species [64]. High power and extended processing durations should achieve significant microbial reduction, leading to increased energy expenditures. Over the past few years, researchers have been looking into combining ultrasound with disinfectants (e.g., chlorine, ozone, and hydrogen peroxide) to lower the cost of microbial inactivation and ultrasound technologies [65]. Existing research has suggested that ultrasonic waves combined with SAEW are capable of effectively reducing microorganisms [66,67]. At present, the combined sterilization treatment of ultrasonic waves and SAEW has been mainly used in aquatic products [68,69]. Based on the bactericidal effect of electrolyzed water and ultrasound on bacteria, the combined treatment of AEW combined with ultrasound is beneficial to accelerate cell death and minimize early damage to bacteria [70], and it is considered as a more environment-friendly novel sterilization technology for minimizing energy consumption, avoiding the use of carbon-intensive chemicals, and reducing toxic release. Thus, this environment-friendly sterilization method has taken on a rising significance.

### 4.1. Synergistic Mechanism of Ultrasonic Waves Combined with Slightly Acidic Electrolyzed Water

Existing research has reported the combined use of ultrasound (US) and slightly acidic electrolyzed water (SAEW) to reduce targeted pathogens over the past few years. Under the mechanical, cavitation, and chemical effects of ultrasonic waves, micro-cracks can be generated in the cell membrane of microorganisms, and the cell membrane permeability can be changed. Cell necrosis or apoptosis is efficiently induced when SAEW is capable of easily reaching the inside of microorganism cells, thus resulting in the increased release of intracellular chemicals [71]. In other words, since ultrasonic bubbles produce high pressures and temperatures, ultrasonication is capable of facilitating the penetration of chemical oxidants into cell membranes, thus increasing the efficacy of disinfectants [72]. The antibacterial effect of ultrasonic waves and SAEW is enhanced, such that SAEW may serve as a liquid culture medium for ultrasonic waves. The cavitation generated by the ultrasonic wave destroys the cell wall of the bacteria in a short period while increasing the contact surface between the SAEW and the bacteria [65].

The sterilization mechanism of ultrasonic waves combined with SAEW treatment can be preliminarily studied by comparing sterilization effects, determining sterilization targets, observing bacterial morphological changes under a scanning electron microscope, and observing bacterial biological characteristics through flow cytometry [73,74]. The results indicate that, unlike single sterilization treatments, combined sterilization treatments can affect the number and quality of bacterial colonies, and simultaneous or continuous exposure to ultrasonic and electrolyzed water pressures may show complementary inactivation mechanisms. In the combination of acidic electrolyzed water and ultrasonic sterilization, the lethality rate is higher than that of ultrasonic waves or acidic electrolyzed water alone [75]. Ultrasound may enhance the microbial sterilization mechanism of acidic electrolyzed water, thus adding convenience for acidic electrolyzed water to penetrate and kill microorganisms during ultrasonication.

**Figure 4:**
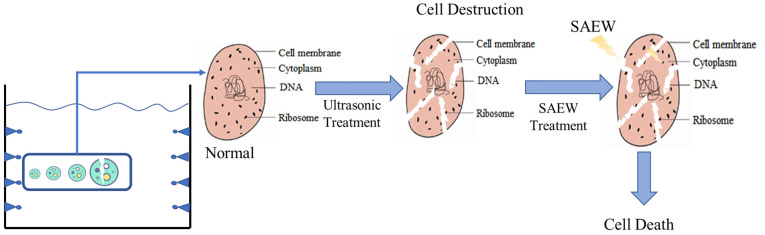
Reaction principle of ultrasonic and slightly acidic electrolyzed water.

In brief, the lethality rate is higher than that of ultrasonic or acidic electrolyzed water alone in the combination of acidic electrolyzed water with ultrasonic sterilization. Ultrasound may enhance the microbial sterilization mechanism of acidic electrolyzed water, thus making it easier for acidic electrolyzed water to penetrate and kill bacteria during ultrasonication. The schematic diagram is shown in Figure 4.

### 4.2. The Impact of Ultrasonic-Assisted Slightly Acidic Electrolyzed Water on Microorganisms

Gram-negative bacteria, particularly *Pseudomonas* and *Shewanella*, are the most common spoilage germs in marine fish (mainly H_2_S-producing bacteria). Lan et al. [76] discovered that the populations of H_2_S-producing bacteria and *Pseudomonas* increased during cold storage, following a similar growth pattern to TVC. The quantity of microorganisms grows as storage duration increases. On day 10, the *Pseudomonas* levels in the distilled water group had 7.0 log10 CFU/g, whereas the ultrasound, SAEW, and ultrasound + SAEW groups had 6.43, 5.49, and 4.89 log10 CFU/g, respectively. Park et al. [77] found that compared with ultrasonic waves or SAEW alone, the synergistic treatment of ultrasonic waves and SAEW could significantly reduce the number of *Escherichia coli* and *Vibrio parahaemolyticus* in fresh-cut herring fillets, but did not affect their sensory quality. The synergistic effect exhibited by the combined treatment of ultrasonic waves and SAEW helps to ensure its sterilization and preservation effect on food, and helps to expand its application range in food sterilization and preservation.

In brief, the synergistic treatment of ultrasonic waves and SAEW and the independent action of the two cause more serious damage to microorganisms, so as to achieve a better sterilization effect.

### 4.3. Application of Ultrasonic-Assisted Slightly Acidic Electrolyzed Water in Aquatic Product Sterilization and Refrigeration

From the above content, it can be seen that both ultrasonic waves and SAEW have a sterilization effect [78].

Li et al. [79] researched the synergistic effect and bactericidal mechanism of ultrasonic wave (US, 200 W, and 30 kHz) and SAEW (60 mg/L, and pH 6.2) treatments, as well as the correlation of mirror carp in cold storage (277.15 K). The study found that using ultrasound in conjunction with SAEW reduced water loss, lipid oxidation, and protein degradation in fresh fish during cold storage. The US + SAEW treatment significantly inhibited microbial growth and endogenous enzyme activity in refrigerated fish, as seen by changes in Pseudomonas properties. A study by Lan et al. [76] introduced that US (20 kHz, 600 W, 600 s) + SAEW (30.0 ± 1.54 mg/L, pH:6.35 ± 0.04) treatment can keep refrigerated seabass fresh. Compared with SAEW or US treatment alone, US + SAEW therapy on sea bass inhibited protein degradation and microbial development, resulting in improved texture and sensory ratings. This combination treatment may extend the shelf life of sea bass by at least 4 days. The findings reveal that the US treatment enhances the ability of SAEW to decontaminate, prevents quality deterioration, and achieves a higher sensory score [80,81].

Accordingly, combined ultrasonic and SAEW treatment has a powerful and efficient bactericidal impact on microorganisms, and the findings support the therapy’s antibacterial mechanism, ability to keep fish fresh, and prolong the shelf life of food.

## 5. Conclusions

Ultrasonic treatment technology is capable of fully retaining the nutrients of aquatic products, and it shows the advantages of high efficiency and low energy consumption, consistent with consumers’ pursuit of a pollution-free, high-quality food consumption concept. SAEW is one of the most promising microbial control fungicides in the food industry, with strong broad-spectrum antibacterial and fungal activity. The synergistic treatment of ultrasonic waves and SAEW can exert a stronger sterilization effect than either treatment alone. Thus, this technology has promising prospects for development in the field of aquatic product processing.

However, the ultrasonic sterilization technology is not mature at present, and faces numerous challenges in terms of large-scale promotion and application. There is a lack of relevant standards and technical parameters for ultrasonic treatment of different aquatic products. When low-intensity ultrasound is employed alone, the killing effect on microorganisms is limited. Moreover, slightly acid electrolyzed water, as a broad-spectrum and efficient bactericide, has been extensively employed to sterilize and disinfect of aquatic products. However, the bactericidal effect of SAEW is correlated with a wide variety of factors (e.g., the mass concentration of available chlorine and treatment time. Relevant parameters should be determined in accordance with the characteristics of different types of aquatic products.

The combination of ultrasonic waves and SAEW with other physical, chemical, and biological technologies will be the future development trend of food sterilization and refrigeration technology to achieve rapid and effective sterilization, environmental protection, food quality improvement, and other purposes. Studying how to optimize the combination of sterilization and refrigeration technologies for different aquatic products and building the sterilization effect data can be beneficial to ensuring food safety and preventing adverse effects on aquatic product quality, while accelerating its industrialization and application.

## Data Availability

The data presented in this study are available on request from the corresponding author.
